# Unveiling gender disparities among medical faculty in a developing country: A case study of a public sector teaching hospital, Peshawar, Pakistan

**DOI:** 10.12669/pjms.41.2.9616

**Published:** 2025-02

**Authors:** Mudir Khan, Brekhna Jamil, Bushra Mehboob, Uzma Bibi

**Affiliations:** 1 Mudir Khan, MBBS, FCPS, MHPE Assistant Professor, Department of Trauma & Orthopedics, Lady Reading Hospital, Peshawar, Pakistan; 2 Brekhna Jamil, BDS, MPH, MHPE Associate Professor, Institute of Health Professions Education & Research, Khyber Medical University Peshawar, Pakistan; 3 Bushra Mehboob, BDS, FCPS, MHPE Assistant Professor, Department of Oral and Maxillofacial Surgery, Peshawar Dental College Riphah International University, Peshawar, Pakistan; 4 Uzma Bibi, M.Phill in Gender Studies Student, Department of Gender Studies, Quaid-e-Azam University, Islamabad, Pakistan

**Keywords:** Administration, Gender disparity, Medical institutes, Policy gap, Teaching faculty, Workplace

## Abstract

**Objective:**

To explore the gendered experiences of medical faculty in tertiary care hospitals in Pakistan.

**Method:**

A qualitative exploratory study was conducted using a semi-structured interview guide in Lady Reading Hospital, Peshawar from 1st April to 30th September 2023. A purposive sampling technique was employed, and data was interpreted using Ackers’s theory of Gendered Organization. Two Focus Group Discussions (FGDs) and six interviews were conducted and transcribed for data analysis. Braun and Clark’s thematic analysis was used for data analysis.

**Results:**

Five themes and twelve sub-themes emerged after data analysis. Most male faculty accepted that gender disparity exists in medical institutions and found an element of ignorance in their responses. On the other hand, most females declared the existence of gender bias in administration, career opportunities, working environment, and basic facilities. Findings revealed the dependency of females on males. A huge gap was also found in policy regarding gender disparity.

**Conclusion:**

Gender disparity exists everywhere because of patriarchal structures. Study unmasked the workplace realities of both genders within medical institutes. Mainly found that females become more victims of this issue. Women lag behind due to the disparity prevalent among both genders.

## INTRODUCTION

Gender Disparity is described as an inappropriate or unfair attitude and behavior towards a particular gender that impacts that gender’s abilities in the workplace.^1^ In Pakistan’s healthcare sector, gender disparity is a critical barrier to the advancement of female medical professionals; despite the fact that 70% of medical students in Pakistan are women, a strikingly low percentage transitions into practicing doctors, highlighting a significant disconnect between education and career progression.^2^ Specific challenges faced by female surgeons include a lack of respect, inappropriate language, inadequate facilities, and limited opportunities for hiring and promotions. These issues are compounded by cultural norms that enforce conservative gender roles, which impose additional barriers on women pursuing careers in medicine.^3^ Even the top residency programs fail to offer paid maternity leaves, daycare centers, support during pregnancy, and support to work mothers. Workplace toxicity and social pressures create difficulty for women to balance their work and home duties, due to which many women are compelled to leave their careers at early stages, hindering the progress of women.^4^

While gender disparity is a global issue,^5^,^6^ its impacts in Pakistan are particularly acute, as evidenced by the country’s ranking of 153rd out of 156 on the gender parity index in the World Economic Forum’s Global Gender Gap Report 2021.^7^,^8^ A study conducted on surgeons of tertiary care hospitals in Pakistan explained the hurdles like lack of respect, use of inappropriate language, lack of facilities in the operation theatre, barriers in hiring and promotions for women, and less presence of role models and mentors throughout their careers.^9^ Another research study concluded that female surgeons are undervalued as leaders, and only one female professor worked in the surgical department, even though 34.4% of residents in the surgery department were female despite the demographic transition “in favor” of women, which is happening globally.^10^ Still, women face the glass ceiling phenomenon when gaining senior positions. Women leadership study in medicine in non-western countries is scanty,^11^ including Pakistan. This research’s objective was to explore the lived experiences of medical faculty members in medical teaching institutes in Pakistan and to contribute to existing literature on women’s leadership in non-western countries. By doing so, the research hopes to inform policy changes and support structures that can help to reduce gender disparities in the medical field, ultimately promoting greater equity and representation for women in healthcare.

## METHODS

The locale selected for the current study was Lady Reading Hospital, Peshawar, one of the largest hospitals in Pakistan, with 32 medical specialties and 2000 beds. The main aim of selecting this locality was to draw the attention of academia and uncover the issues and challenges of gender. To fulfil the purpose of the study, a Qualitative Exploratory research design with a narrative approach was used, which is significant in generating data, including the assumptions and value of the study researcher.^12^

A non-probability, purposive sampling technique was used to add an under-researched population in their context. In qualitative research, the sample size is determined by data saturation. In a homogenous sample, saturation is reached at a sample size of 12-15.^13^

### Ethical Approval:

The ethical approval for the study was obtained from Khyber Medical University, Ref No:1-11/IHPER/MHPE/KMU/23-37, dated June 15, 2023.

A total of 27 participants were selected. A sample size of six respondents was selected, including three males and three females, for individual interviews and two focused group discussions (FGD), one with nine males and the other with twelve females done for in-depth data.

### Inclusion and Exclusion Criteria:

The inclusion criteria were faculty members at the assistant professor and above, including both genders with at least three years of teaching experience at this institute. While locum, visiting faculty, and non-willing participants were excluded.

Demographic characteristics (Gender, designation, specialty) were recorded. The study was conducted for six months, from 1st April to 30th September 2023. A semi-structured interview guide was designed based on twelve open-ended questions by considering the feminist theoretical lens, the theory of gendered organization.

**Fig.1:**
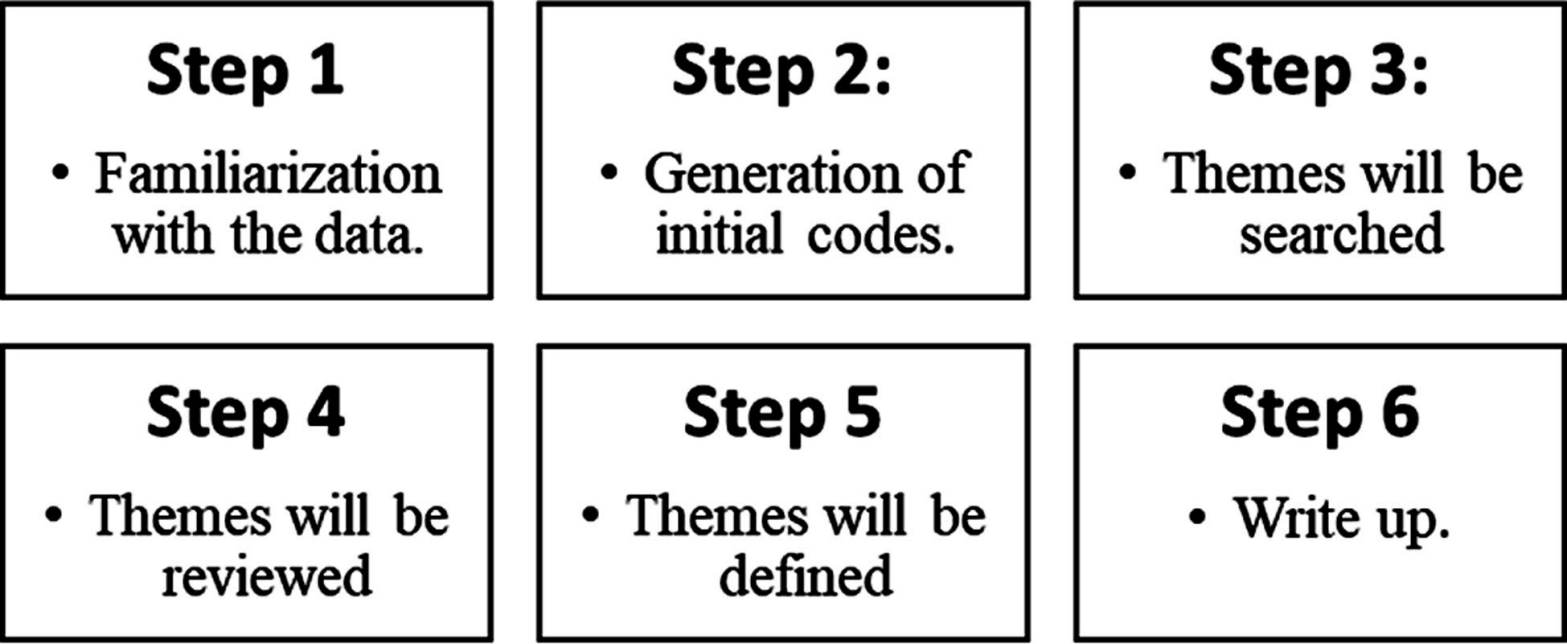
Braun and Clark’s thematic analysis.

A pilot study was conducted before the major study. Informed consent and Permission to audio-record the conversation was obtained, and the participants were ensured confidentiality and anonymity. The data was collected until saturation was achieved. The saturation point was when no new themes or insights emerged from the interviews, indicating that enough information had been gathered to understand the subject matter thoroughly. Braun and Clark’s thematic analysis was followed for data analysis (Fig.1).

## RESULTS

Table-I provides demographic details, while Table-II tabulates the main themes and sub-themes.

**Table I:** Demographic details of the participants.

S.No.	Designation	Gender	Department	Years of experience
***Interviews participants***				
1	Assistant Professor (AP)	Male	Cardiology	6
2	AP	Male	Peads surgery	3.5
3	AP	Male	General surgery	4
4	AP	Female	Cardiology	5
5	Associate professor	Female	Gynecology	8
6	AP	Female	Anesthesia	3.5
***Focused group discussion (male)***				
1	AP	Male	Thoracic surgery	7
2	AP	Male	Orthopedic surgery	5
3	AP	Male	Pulmonology	4
4	AP	Male	Anesthesia	3.3
5	AP	Male	Anesthesia	3.5
6	AP	Male	Endocrinology	4
7	AP	Male	Anesthesia	3
8	AP	Male	Vascular surgery	3
9	AP	Male	G. Surgery	4
***Focused Group Discussion (Female)***				
1	AP		Paeds medicine	4
2	AP		Anesthesia	4
3	Professor		Gynecology	20
4	Associate professor		Gynecology	13
5	AP		Radiology	4
6	AP		Radiology	7
7	AP		Gynecology	5
8	AP		Paeds medicine	7
9	Associate		Pulmonology	18
10	AP		Paeds medicine	6
11	AP		Anesthesia	9
12	AP		G. Surgery	5

**Table-II:** Issues and Challenges of faculty members considering Gender-based disparity.

Themes	sub-Themes
Gender disparity and cultural norms	Meaning of Gender disparity
	A decided journey
	Transferred unfairness and culture favors
	Male grievances: A discrimination but beneficial
Policy Gapes	Lack of policies
	Digested inequalities
	Opportunities vs. skills
Lived Workplace Experiences	Workplace Narratives: Interaction with Fellow beings
	Unfairness under the surface of fairness
	Appreciation hurts, though why?
Navigating the dominance of authority	Administrative disparities
	The will of boss is everything

### Theme-1: Gender disparity and cultural norms:

In this theme, the participants mentioned the differing perspectives on gender disparity as observed by respondents. It indicated that both men and women have unique viewpoints on the issue, which suggests that their experiences and societal roles shape their opinions. Additionally, the participants emphasized that gender disparity is pervasive, meaning it is not limited to specific areas but is evident in both private settings, like homes and personal relationships, and public domains, such as workplaces and communities. This highlights the widespread nature of the issue and the need for awareness and action across various contexts. Their division in roles traditionally exists in both the public and private domains. This dichotomous division makes women still dependent on men.

*“Although I am working, but still, I am not fully independent. My job depends on my husband’s job*.

*When he wants to shift at some other place. I have to leave my job.”- **(Female Respondent 2)***

*“Everyone’s thought and views depiction is based on the fact that he is a male or she is a female**” (Female respondent-4)***

*“Unequal treatment or bad treatment based on gender is called gender disparity.”*

### - (Female respondent 1)

Female respondents noted that males often exhibit bias due to their conservative thinking and backgrounds in rural areas. Institutions operate within the frameworks of society, where unwritten rules often have a greater impact than formal laws. Culture permeates every aspect of life and shapes behavior and perceptions.

*“The reason is that they have been brought up like that, that a female should not refuse them, they don’t want the female to be dominant”-* ***Female respondent 3***

*“We are seeing like there are more female students in medical colleges, but when it comes to practical life, males are in greater proportion because of the fact that when females get married, they are not allowed to work, or they’re having no support for their family.”*

### - Female Respondent-in FGD

The participants expressed the opinion that everyone is affected by gender disparity. Female colleagues often receive favoritism, and when replacements for these female employees are needed, male doctors typically work during night shifts. Women tend to receive support primarily due to their gender and the life challenges they face.

*“Once, I was on my night shift because of an emergency case. But I couldn’t do it because my children were alone at home. So, my male colleagues show positive response and replace my duty”*- ***Female respondent-2***

### Theme-2: Policy Gaps:

Respondents expressed their desires for specific policy recommendations. While our study revealed that policy implementation in medical institutions is inadequate, many respondents were unaware of existing policies related to maternity and paternity leave.

*“We cannot stop a female giving birth, asking them to come and work. When she cannot able to get up early, it creates problems. In this crucial time period husband should be there, So, paternity and maternity leave should be there, but it is not gendered as it is their right”- **Male respondent from FGD***

### Theme-3: Lived Workplace Experiences:

Female respondents highlighted a lack of opportunities, while male respondents attributed issues to a perceived lack of skills and capabilities. Furthermore, women often contribute to the inequalities and injustices present in academia by remaining silent about the micro-level disparities they face. Female respondents support the existence of gender disparities, while male respondents tend to take this issue less seriously.

In their concluding remarks, female respondents expressed both positive and negative interactions with their male counterparts. They noted that they receive support from their male colleagues, especially during challenging times. Male respondents, on the other hand, stated that the presence of females does not significantly impact them, as they view this situation through a lens of professionalism.

*“What I have observed is that male colleagues are friendly with each other. Yeah, but when we come, they don’t want us to reach their places.” **Female respondent 2***

*“We have been (pause), we have brought up like this, you know, means that females cannot do this, if we are given confidence or equal opportunities, our parents should say that you can also do that, so, we will be able to do any kind of job” **Female respondent-2***

*“They think that females are more fragile, sensitive emotionally, and they cannot cope with the most difficult situations” **A female respondent from FGD***

*“We usually preferred males because if somebody is a female definitely, she is going to have children and have to fulfill family commitments. Except from Gynecology department majority are males”- **Male respondent 2***

The female participants mentioned that verbal praise has a detrimental effect on them, as they sometimes intertwine it with harassment, creating an uncomfortable and damaging experience. Male respondents thought that women often receive more appreciation and leniency in various situations than their male counterparts, resulting in fewer penalties and criticisms.

*“It hurts me when verbal reward becomes a punishment for us. We timidly reject it”- **Female respondent 2***

*“Female respondents feel isolated and excluded at the workplace because of their familial responsibilities, workload and due to lack of comfortability.” **Male** **Respondent from FGD.***

### Theme-4: Navigating the dominance of authority:

Administrative posts are termed “masculine” because mostly males are seen in them. Both males and females share the same view that most administrative posts are occupied by males, and the reason behind this is the assumption that females can’t manage administrative tasks efficiently because of a perceived lack of ability and experience.

*“Gender biasness exists in administrative domain. Administrative posts are given to male gender because our society thinks that they are more capable of doing administrative tasks”- **Female respondent from FGD***

The responses from male and female participants revealed contrasting perspectives. Most male respondents indicated that having female bosses tends to benefit them more, while female respondents noted that male bosses are generally cooperative with them.

*“Yes, that would be definitely if you are a male, a male boss and you have got two trainees, one male and the other is female and both have done their jobs, you tend to give a bit of more credit to the female trainee because that is something that is natural and obvious. And yes, if you have a female boss, then in this case appreciation will be given to male. So, it depends upon the gender of the boss who is giving the appreciation”- **Female respondent from FGD***

## DISCUSSION

The current study highlights the lived experience of gender disparity among women working in clinical fields. The theme “Gender disparity and cultural norms,” as explained by interviewees, encompassed unequal treatment, biased behavior, and limited opportunities based on gender. Literature has mentioned that women have a main role in the family, so they are not given equal opportunities relative to men. Female respondents highlighted discrimination and bias in medical professions, especially in surgical careers. One of the studies conducted in a tertiary care hospital in Pakistan also found the same findings.^8^

The discussion on gender disparity highlighted the predetermined societal paths for men and women. Traditional roles, reinforced by a male chauvinistic approach, devalue women’s work, impacting career choices.^14^ Despite women’s increasing representation, universities and medical professions remain male-dominated, affecting academic rankings. Literature shows that women are mostly under-represented at the senior level.^15^ Despite half of the population, women are still underrepresented among practicing physicians and faculty leadership roles in North America, except in dermatology.^16^ The dichotomous journey, burdensome for both genders, perpetuates unequal treatment in public and private domains, influencing career selection and workplace valuation of women’s contributions.

Female fear of repercussions in male-dominated environments leads to compliance, particularly in departments where bias is prevalent. Deeply ingrained cultural norms in Pakistan discourage women from practicing medicine, with biases attributed to being perceived as the ‘other. Male medical faculty allege favoritism towards female students in grading and authoritative treatment. Male respondents see this as gender bias, while female respondents perceive it as necessary support due to societal expectations and biological differences.

In theme two a policy gap was found in gender-neutral regulations. Participants advocate for gender equity policies, anti-discrimination measures, and fixed maternity and paternity leave policies. The absence of a daycare center for working mothers and dissatisfaction with the current maternity leave policy was also discussed, emphasizing the need for institutions to provide supportive environments for both genders. The literature also supported the opinion that females are getting fewer opportunities.^5^,^8^,^17^–^19^ On the other hand, the full professor level in Turkey has a shockingly high proportion of women due to the country’s highly transparent promotion procedure.^20^ The results are noteworthy: from 2009 to 2014, 22% more female full professors worked at academic medical facilities in the United States. Only seven of the 50 universities financed by the National Institutes of Health had more than 20 % female department leaders.^21^

The study respondents shared personal experiences of facing disparity in time allocation, invisible workload, and hurdles in career advancement. They felt discriminated against by their male colleagues, support staff, and administration, which men predominantly occupied. The literature reflected the mixed situation of workplace gender discrimination with regard to time allocation and invisible workload.^19^ Ward and Sloane explained that females have less satisfaction in the workplace than their male counterparts.^22^ Female faculty, as compared to male faculty, feel isolated in the chilly climate.^23^

The study participants also explored the flawed concept of binary gender distinctions, highlighting how women are often labelled as the “other.” Women shared experiences of feeling unprotected and isolated in public spaces, expressing concerns about harassment. The literature also supports women’s feelings of limited safety in public spaces and being considered “other”.^24^,^25^ This study also revealed that the boss’s will, influenced by their gender, determines appreciation or discrimination in the workplace. Female respondents note that the boss’s gender plays a crucial role in the distribution of credit and favoritism. Male respondents acknowledge the impact of having a female boss and express contentment when making merit-based decisions. It underscores the powerful role of individual willpower in navigating systemic injustices within the medical faculty. Similar results are reported in other fields.^26^ Social issues faced by women physicians, & Barriers to their success in academic medicine were earlier highlighted.^27^

### Limitations:

This research study has some limitations that should be acknowledged. Firstly, the sample size is relatively small and restricted to only one medical teaching institution. This geographic limitation means that the findings may not capture the diversity of experiences and perspectives found in other institutions or regions. Variability in teaching methods, faculty expertise, and student demographics among different medical schools could influence the applicability of the results beyond our study site. While the study provides valuable insights, caution must be taken when attempting to apply these findings universally to other educational settings. Overall, these limitations highlight the need for further research across diverse contexts to validate and expand upon the conclusions drawn in this study.

## CONCLUSIONS

The findings of this study highlight the pervasive nature of gender disparity within medical institutions, revealing the underlying cultural and structural challenges that contribute to these inequities. The evidence suggests that women face significant barriers in their professional advancement, influenced by ingrained patriarchal norms that persist in these environments.

This disparity affects the career trajectories of female professionals and impacts the overall quality of care and innovation within the medical field. When diverse voices and perspectives are sidelined, the potential for holistic and effective healthcare solutions diminishes. Addressing gender disparity is not just a moral imperative but also a significant opportunity for growth and improvement within the medical sector. Only by recognizing and actively combating these inequalities enable us to create a more equitable and effective healthcare system for all.

### Recommendations:

The findings of this study suggest that medical institutions should develop and implement comprehensive policies aimed at promoting gender equality. This includes ensuring equitable hiring practices, transparent promotion criteria, and support systems for all genders. Mentorship programs should be initiated connecting female faculty members with experienced professionals to provide guidance, support and encouragement. Regular training sessions should be conducted on gender sensitivity and workplace equality, more over the institutes should adopt flexible working arrangements and provide support services such as paid maternity leave, and childcare facilities**.** These recommendations aim to create a more equitable and supportive environment for all faculty members, helping to bridge the gender gap in medical institutions in Pakistan.

### Authors’ Contribution:

**MK:** Conceived, designed, and did the analysis, writing of the manuscript & responsible and for the accuracy of the study.

**BJ:** Did the review and final approval of the manuscript.

**BM:** Helped in the conceptual design of the study, edited the manuscript, and gave final approval of the manuscript.

**UB:** Did transcription of data and literature review.
